# Consumers’ Use of UMLS Concepts on Social Media: Diabetes-Related Textual Data Analysis in Blog and Social Q&A Sites

**DOI:** 10.2196/medinform.5748

**Published:** 2016-11-24

**Authors:** Min Sook Park, Zhe He, Zhiwei Chen, Sanghee Oh, Jiang Bian

**Affiliations:** ^1^ School of Information Florida State University Tallahassee, FL United States; ^2^ Institute for Successful Longevity Florida State University Tallahassee, FL United States; ^3^ Department of Computer Science Florida State University Tallahassee, FL United States; ^4^ Department of Health Outcomes and Policy University of Florida Gainesville, FL United States

**Keywords:** controlled vocabulary, consumer health vocabulary, concept coverage

## Abstract

**Background:**

The widely known terminology gap between health professionals and health consumers hinders effective information seeking for consumers.

**Objective:**

The aim of this study was to better understand consumers’ usage of medical concepts by evaluating the coverage of concepts and semantic types of the Unified Medical Language System (UMLS) on diabetes-related postings in 2 types of social media: blogs and social question and answer (Q&A).

**Methods:**

We collected 2 types of social media data: (1) a total of 3711 blogs tagged with “diabetes” on Tumblr posted between February and October 2015; and (2) a total of 58,422 questions and associated answers posted between 2009 and 2014 in the diabetes category of Yahoo! Answers. We analyzed the datasets using a widely adopted biomedical text processing framework Apache cTAKES and its extension YTEX. First, we applied the named entity recognition (NER) method implemented in YTEX to identify UMLS concepts in the datasets. We then analyzed the coverage and the popularity of concepts in the UMLS source vocabularies across the 2 datasets (ie, blogs and social Q&A). Further, we conducted a concept-level comparative coverage analysis between SNOMED Clinical Terms (SNOMED CT) and Open-Access Collaborative Consumer Health Vocabulary (OAC CHV)—the top 2 UMLS source vocabularies that have the most coverage on our datasets. We also analyzed the UMLS semantic types that were frequently observed in our datasets.

**Results:**

We identified 2415 UMLS concepts from blog postings, 6452 UMLS concepts from social Q&A questions, and 10,378 UMLS concepts from the answers. The medical concepts identified in the blogs can be covered by 56 source vocabularies in the UMLS, while those in questions and answers can be covered by 58 source vocabularies. SNOMED CT was the dominant vocabulary in terms of coverage across all the datasets, ranging from 84.9% to 95.9%. It was followed by OAC CHV (between 73.5% and 80.0%) and Metathesaurus Names (MTH) (between 55.7% and 73.5%). All of the social media datasets shared frequent semantic types such as “Amino Acid, Peptide, or Protein,” “Body Part, Organ, or Organ Component,” and “Disease or Syndrome.”

**Conclusions:**

Although the 3 social media datasets vary greatly in size, they exhibited similar conceptual coverage among UMLS source vocabularies and the identified concepts showed similar semantic type distributions. As such, concepts that are both frequently used by consumers and also found in professional vocabularies such as SNOMED CT can be suggested to OAC CHV to improve its coverage.

## Introduction

### Background

There is a widely known language gap between health consumers and health care professionals [1-3]. This gap may hinder effective communication between the 2 groups [4-7]; thus, impacting consumers’ health information seeking [3,8,9] and subsequent decision making regarding their health issues [10]. To assess the gap, Roberts and Demner-Fushman [11] used a variety of natural language processing (NLP) techniques to analyze the difference between health questions asked by consumers and health professionals in different online question and answer (Q&A) sites (eg, Yahoo! Answers, and WebMD). They found that consumer questions tend to contain more misspelled medical terms, have longer background information, and resemble open-domain language more closely than texts written by professionals. One major aspect of the gap is the difference in medical vocabulary used by consumers and health professionals. Zeng and colleagues [12] observed that when searching online health information, using only consumer terms leads to poor information retrieval results. Plovnick and Zeng [13] later reformulated consumers’ health queries with professional terminology and about 40% of reformulated queries yielded better search performance.

To bridge the vocabulary gap between health professionals and consumers, early researchers have collected and analyzed diverse textual data generated by consumers to identify medical terms used by consumers. Brennan and Aronson [14] used the MetaMap tool to extract salient concepts in nursing vocabularies from consumers’ email messages. Smith and collegues [15] also used MetaMap to successfully identify the Unified Medical Language System (UMLS) concepts used by consumers in their email messages submitted to University of Pittsburg Cancer Institute’s Cancer Information and Referral Service. These studies aimed to bridge the vocabulary gap between health professionals and consumers by identifying frequently-used consumer health terms that are relevant in developing consumer-oriented health information applications and linking free text to complex clinical knowledge resources. These *ad hoc* studies represent early efforts in bridging the vocabulary gap.

A controlled vocabulary is “an organized arrangement of words and phrases used to index content and/or to retrieve content through browsing or searching[16].” In an effort to formalize consumer vocabulary for various applications, a controlled vocabulary called Open-Access Collaborative Consumer Health Vocabulary (“OAC CHV,” “CHV” for short) was recently developed as a collection of expressions and concepts that are commonly used by ordinary health information users [17]. Moreover, CHV has been integrated in the largest medical terminological system–the UMLS, which has mapped terms from different source vocabularies with the same meaning into the same concept by the United States National Library of Medicine (NLM). As such, consumer terms are connected to their corresponding professional terms in professional vocabularies such as SNOMED Clinical Terms (SNOMED CT). With CHV in the UMLS, one can translate a sentence with consumer terms to a sentence with professional terms in an automated fashion.

Domain coverage—the extent to which a controlled vocabulary covers the intended domain—is one of the most desired properties for a controlled vocabulary [18]. The usability and the overall structure of a controlled vocabulary heavily rely upon its coverage [19]. Traditionally, controlled vocabulary development takes a top-down approach, which reflects a group of experts’ knowledge in the respective subject matter [20,21]. For the development of CHV, however, a bottom-up approach was taken, emphasizing 2 fundamental properties: (1) CHV should capture actual consumers’ terms and expressions that reflect their health information needs, and (2) the expressions should be familiar to and used by consumers [17].

To keep up with continuous evolution of medical knowledge, CHV needs to be updated and maintained by incorporating new, consumer-provided terms and expressions [17,22-24]. Existing studies have shown promising results in discovering consumer terms for CHV from social media, in particular. Vydiswaran et al [7] applied a pattern-based text mining approach to identify pairs of consumer and professional terms from Wikipedia. Hicks et al [25] analyzed consumer messages exchanged in Twitter in order to evaluate terms related to gender identification on intake forms. Doing-Harris and Zeng-Treitler [24] developed a computer assisted CHV update system, which can automatically identify prospective terms from social media. Identifying terms used by consumers in consumer-generated text in aggregate fashion can account for the variability of lay health language. These terms can be used to refine and enrich CHV [17].

Consumers, however, may also learn and use professional terms [17,24,26]. In this sense, medical terms that are familiar to consumers and are already established in other controlled vocabularies could be used to improve the coverage of CHV. Term reuse is a principle and best practice in ontology/terminology development as it promises to support the semantic interoperability and to reduce engineering costs [27]. Researchers have previously developed semi-automated methods to facilitate systematic term reuse. He et al [28] developed a topological-pattern-based method to identify terms from UMLS source vocabularies to enrich SNOMED CT [28,29] and National Cancer Institute Thesaurus (NCIt) [30].

However, this method cannot be directly applied to CHV, because it does not have hierarchical relationships (eg. parent-child relationship) that are necessary to construct topological patterns [28-30]. Recently, Chandar et al [31] introduced a similarity-based term recommendation method that represents n-grams extracted from the free-text eligibility criteria of clinical trials as a set of linguistic and contextual features. SNOMED CT terms are clustered with K-means clustering. The new terms are ordered by their distance to the nearest cluster centroid, representing their similarity to existing SNOMED CT terms. This method performed well on the corpus of free-text clinical study eligibility criteria, because they are mostly short and partial sentences written by health professionals with fruitful medical terms and little noise. It has yet to be tested on free-form consumer text that typically contains lengthy sentences and lay terms.

Most previous studies concerning CHV development concentrated on the identification of new terms used by consumers [17,22-24]. To the best of our knowledge, no prior studies have conducted in-depth assessment of the coverage and popularity of medical concepts in user-generated documents on social media. In this respect, there is a need to understand consumers’ use of terms in existing controlled vocabularies, and to perceive if there is the potential to improve CHV by incorporating health-related concepts used by consumers that are covered by professional vocabularies. In this study, therefore, we performed such an analysis in order to assess consumers’ use of medical concepts on social media postings pertaining to health concerns and to evaluate how many popular consumer terms have been included in the existing source vocabularies of the UMLS [32].

In this study, we focus on diabetes, which is recognized as one of the most important public health problems with escalating health concerns by the World Health Organization (WHO) [33]. Diabetes caused 1.5 million deaths in 2012 alone. It is known to cause disability and an array of serious health issues such as hypertension, nephropathy, and stroke [34]. Global diabetes cases skyrocketed from 108 million in 1980 to 422 million in 2014. The number of diabetes onset will likely reach 700 million by 2025 [35]. Diabetes and its complications not only impair population health but also impose substantial economic burdens on patients, their family, and the society [33].

In this study, we collected diabetes-related consumer-generated blog postings from Tumblr and diabetes-related questions and answers from Yahoo! Answers. We carried out text mining to identify UMLS concepts from our datasets. Thus, we formulated the 2 research questions (RQs): (1) To what degree do the concepts from UMLS source vocabularies cover the concepts used by consumers describing their diabetes-related concerns on health postings of social media, especially blogs and social Q&A? Which concepts do or do not overlap? (2) To what degree are the UMLS semantic types applicable to analyzing the concepts used by consumers when describing their diabetes-related concerns in social media, especially blogs and social Q&A? Which semantic types are observed?

In the first research question, we evaluated the coverage of all of the 178 English source vocabularies of the UMLS in our 2 datasets from Tumblr and Yahoo! Answers. In the second research question, we analyzed the semantic types of the UMLS concepts identified in our datasets.

The current study mainly investigated the overlap between consumer concepts from social media and professional concepts in the UMLS. Indeed, consumers often proactively seek and share online health information on social media [36,37]. Their use of professional terms could be sophisticated covering both laypersons’ expressions and medical terminologies. In fact, not only consumers but also health care professionals have actively participated in creating health postings in social media [38,39]. Their use of terms in social media, however, is likely to be more consumer/patient-centric for health education and promotion to the public. The comparative analysis of the concept coverage between consumers and professional vocabularies in social media may be helpful in understanding the scale of the phenomenon. The comparison will also help yield insights into the nature of the vocabulary gap, which will contribute to the consistent development of the CHV. The current study, in particular, could shed light on how much social media users use existing terms in UMLS source vocabularies on the web. At the same time, findings from the current study could inform the feasibility of leveraging existing UMLS source vocabularies to enrich the CHV.

### The Unified Medical Language System

The UMLS, maintained by the NLM of the National Institutes of Health, is the largest biomedical terminological system. Its 2-level structure consists of Metathesaurus and Semantic Network. The UMLS Metathesaurus is “a large, multi-purpose, and multi-lingual thesaurus that contains millions of biomedical and health related concepts, their synonymous names, and their relationships” [40]. The UMLS Metathesaurus integrates more than 9.1 million terms from over 170 English source vocabularies into 3.1 million medical concepts (2015AA version). Besides English, the UMLS also contains source vocabularies in 20 other languages. The UMLS has integrated most of the well-designed and well-maintained medical terminologies such as SNOMED CT, the International Classification of Diseases 9^th^ Revision, Clinical Modification (ICD-9-CM), NCIt, and RxNORM. SNOMED CT is the most comprehensive and precise clinical terminology in the world with over 310,000 active concepts [41]. ICD-9-CM is used primarily to encode the diagnoses and procedures for billing purposes [42]. RxNORM, on the other hand, normalizes names of all clinical drugs available on the US market and their links to many of the drug vocabularies commonly used in pharmacy management [43]. Most significantly, the terms with the same meaning are mapped to the same concept in the UMLS. Due to its native term mapping, the UMLS is a valuable resource for supporting interoperability and translation in biomedicine [32]. The NLM releases a new version of the UMLS twice a year.

The UMLS semantic types represent “a set of broad subject categories that provide a consistent categorization of all concepts represented in the UMLS Metathesaurus” [44]. Each concept in the UMLS is assigned 1 or more semantic types. In the 2015AA version of the UMLS, there are a total of 127 semantic types, describing concepts at the levels of entity and event. Entities include physical objects such as organism, anatomical structure, and substances. Events describe activities, phenomenon, and processes. For example, the semantic type “Disease or Syndrome” categorizes a set of concepts in the UMLS that indicate “a condition which alters or interferes with a normal process, state, or activity of an organism.”

### Consumer Health Vocabularies and Their Use in Consumer-Oriented Health Applications

OAC CHV has been used in various health-related applications to improve patients’ access to health information. Zeng et al developed a translator specifically to convert texts in electronic health records to consumer-friendly text in patient health records by replacing UMLS terms to their corresponding OAC CHV terms [45]. Many UMLS concepts have one to one match with OAC CHV concepts. All the OAC CHV concepts have predefined consumer-friendly display names. Besides OAC CHV, other proprietary consumer health vocabularies have been developed. For example, Apelon has developed a CHV and has mapped their CHV terms to corresponding clinical concepts in SNOMED RT (an earlier version of SNOMED CT, developed by College of American Pathologists), ICD-9-CM, and Physician’s Current Procedural Terminology (CPT) administrative codes. The CHV of Apelon has been used in various applications, such as consumer health data entry, patient results reporting clinical note translation, and Web-based information retrieval [46]. Mayo Clinic also developed their own consumer health vocabulary, which has a rich content of disease concepts as well as genetic and non-genetic risk factors to diseases [8]. In this paper, we used OAC CHV because it is the only publicly available consumer health vocabulary that we have access to (through the UMLS).

## Methods

### Data Collection

2 types of social media were analyzed in the current study, namely blogs and social Q&A, as they allow consumers to generate and freely exchange health information in text format. Health-related blogs are one of the most popular social media venues for health information distribution. Bloggers typically describe their personal experiences with diseases along with their encounters with health care professionals [47]. Health care professionals also create blogs for sharing their medical knowledge and information with patients [48]. Blogs have also been widely used for health promotion and education as a collaborative tool for both consumers and health care professionals [49-51]. On the other hand, social Q&A is an online community-based Q&A service where people gain knowledge through raising questions and receiving answers from others who willingly share their knowledge, experiences, and opinions regarding a wide range of topics including health. Social Q&A is considered to be a knowledge-shaping sphere for laypeople [52]. Consumers are motivated to use social Q&A because their searches on web search engines with short queries that are not fully expressive often fail in retrieving useful information for their specific problems, while social Q&A allows them to ask questions in natural language and in full sentences [11]. For data collection, we used 2 datasets: (1) Tumblr, a popular blogging service; and (2) Yahoo! Answers, a social Q&A service in North America.

Tumblr and Yahoo! Answers were chosen for the current study due to their popularity and the convenience of using their Application Program Interfaces (APIs), which allowed us to collect data automatically from these sites. Also, both Tumblr and Yahoo! Answers do not limit the number of words in postings. As such, their users can elaborate their health concerns and information on postings with sufficient details, thereby providing us ample opportunities to extract and analyze relevant concepts from the postings.

Tumblr is one of the fastest-growing blog sites with nearly twenty-fold increase in the number of blogs from October 2012 to October 2015 [53]. It launched relatively late in the market compared to other sites such as WordPress and Blogger, but is recognized as one of the best blog sites due to its ease of setup, stylish interface design, and micro-blogging support [54,55]. It has over 227 million blogs and 37 million unique visitors as of February 2016 [53]. From Tumblr, we collected a total of 3711 English text blogs with a tag related to “diabetes” (eg, “diabetes,” “diabetes mellitus,” and “Type 2 diabetes”) posted between February and October 2015.

Yahoo! Answers is one of the most popular social Q&A sites with approximately 5.6 million visitors per month as of February 2016 [56]. From Yahoo! Answers, we garnered a total of 58,422 questions and associated answers between 2009 and 2014 in the diabetes category of Yahoo! Answers. During data analysis, we carried out text mining with questions and answers (specifically, best answers) separately, because the information in questions and answers could be different. Questions could capture health concerns and associated problems, while answers could mainly discuss information resources intended to solve the problems. It is important to note that 1 question may have more than one answer. In this study, we limited answers to the one selected as the best answer by the questioner. The data collected from Yahoo! Answers were separated into questions and answers in the subsequent analyses.

### Units of Analysis

Once we collected text data from Tumblr and Yahoo! Answers, we mined the text data for “concepts,” a unit of understanding which represents a fundamental component of terminology [57] or unit of meaning in an ontology [31]. Concepts are different from “terms” in that a term refers to an entity or “physical object” written or spoken in text to represent a concept or thought [58]. In the UMLS, a term is described as a “word or collection of words comprising an expression,” which indicates a class of all lexical variants (eg, “eye,” “Eye,” “eyes”) [59]. The UMLS assigned each term an atom unique identifier (AUI) and grouped the terms with the same meaning into a concept with a concept unique identifier (CUI). We also analyzed the semantic types of the extracted concepts in order to understand the broad semantic categories of the terms that are frequently used by consumers.

### Textual Data Processing

We used a widely adopted biomedical text processing framework Apache cTAKES™ [60] and its extension YTEX [61] to identify UMLS terms in our datasets. Apache cTAKES is designed as a natural language processing (NLP) system for extraction of information from the free-text data available in electronic medical records (EMRs). It provides an agile and flexible platform based on the Unstructured Information Management Architecture (UIMA) and a rich NLP library. YTEX, a module of cTAKES, provides Word Sense Disambiguation (WSD), data mining and feature engineering functionalities. We mainly used the WSD function of YTEX to recognize the most possible UMLS concept when a term in the free text can be matched to multiple ambiguous concepts. We used the 3.2.2 release of cTAKES and YTEX with the default workflow configuration named “Aggregate Plaintext UMLS Processor.”

**Figure 1:**
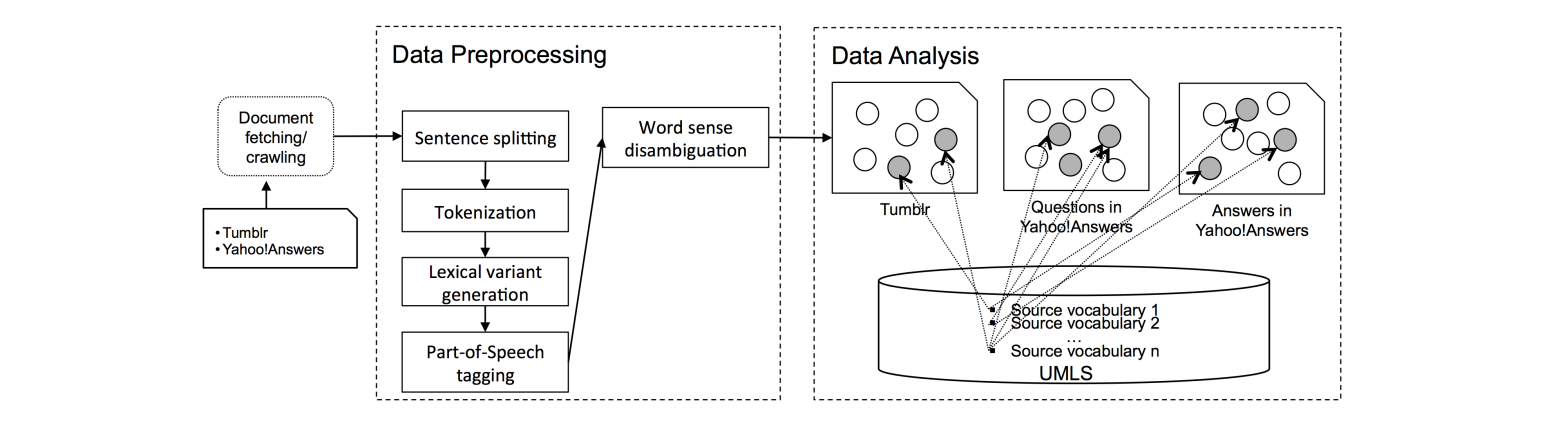
Conceptual framework of the study. Dots refer to concepts extracted from the dataset and gray dots refer to concepts mapped to the concepts in one of the UMLS source vocabularies.

Figure 1 illustrates our overall analysis process. First, each document is a blog posting from Tumblr, a question or an answer from Yahoo! Answers. Each blog posting may consist of 1 or more sentences. Then, cTAKES detected and split each document into individual sentences using the sentence detector of OpenNLP [62,63], with the default configuration for English text. For each sentence, cTAKES performed tokenization with the default tokenizer of the OpenNLP, lexical variant generation using the lexical tool provided by the United States National Library of Medicine with the default configuration. Then, cTAKES performed Part-Of-Speech (POS) tagging using the POS tagger in OpenNLP with the information entropy-based model for English to generate the candidate terms for further processing. Then, YTEX matched the candidate terms with all the possible UMLS terms, which were preloaded from the MRCONSO table of the UMLS 2015AA release. We then stored the matching results to a MySQL database. For each candidate term, there may be 0, 1, or more matching UMLS terms with different semantics. To identify terms with reasonable semantics, we used YTEX to perform word sense disambiguation (WSD), in which the intrinsic information content (IC) measure is used as the semantic similarity metric with a window of 50 words as the context for WSD. The intrinsic information content is a measure of concept specificity computed from the structure of the taxonomy in a biomedical terminology and does not rely on the term frequency in the corpus. The details of the intrinsic IC measure can be found in Garla et al [64]. Finally, all the UMLS terms in each record were extracted with a UMLS CUI.

### Concept Coverage Analysis

We first analyzed the basic characteristics of the overall concept coverage across our datasets collected from Tumblr and Yahoo! Answers: (1) blog postings from Tumblr, (2) questions in Yahoo! Answers, and (3) answers in Yahoo! Answers. We then analyzed the coverage of each source vocabulary in the UMLS across the datasets. SNOMED CT and CHV are the 2 vocabularies with the highest concept coverage in our datasets. Thus, we conducted a concept coverage analysis of SNOMED CT and CHV based on our datasets. We also analyzed the semantic types of the concepts identified from our datasets.

## Results

### Aggregate Characteristics of the Datasets

We identified 2415 UMLS concepts from blog postings, 6452 UMLS concepts from questions, and 10,378 UMLS concepts from answers. Table 1 shows the total number of documents and sentences in our datasets (ie, blog postings, questions, answers). These numbers were compared to the “# with UMLS concepts,” the unique number of documents and sentences containing the identified UMLS concepts. Note that we can only extract concepts that are presented in UMLS. Thus, the total number of concepts in our datasets (which can include concepts that are not in UMLS) is not provided in Table 1.

There was a noticeable variation across the datasets. Over 80% of the documents from questions and answers contained 1 or more UMLS concepts whereas less than half of the documents from blogs did. Over half of the sentences from questions and answers contained at least 1 UMLS concept, while only 27% of those from blog posts contained at least 1 UMLS concept.

**Table 1:** Basic characteristics of UMLS concept coverage in our datasets.

	Tumblr		Yahoo! Answers			
	Blog postings		Questions		Answers	
	Total #	# with UMLS concepts	Total #	# with UMLS concepts	Total #	# with UMLS concepts
Document	3711	1388 (37.4%)	58,422	51,850 (88.8%)	58,422	51,550 (88.2%)
Sentence	47,413	12,802 (27.0%)	249,013	142,802 (57.3%)	348,793	216,736 (62.1%)
Concepts	–	2415	–	6452	–	10,378

### Coverage by the UMLS Source Vocabularies

The concepts in the blogs were covered by 56 UMLS source vocabularies, while those in questions and answers were covered by 58 source vocabularies. Table 2 illustrates the top 20 most covered UMLS source vocabularies (The full names and the version information of the source vocabularies can be found in the Multimedia Appendix 1 Table-A1). SNOMED CT was dominant across all our datasets, ranging from 84.9% to 95.9%. It was followed by CHV (between 73.5% and 80.0%) and MTH (between 55.7% and 73.5%). Other source vocabularies within the top 10 for all of our datasets are: NCIt, Medical Subject Headings (MeSH), Computer Retrieval of Information on Scientific Projects Thesaurus (CSP), Library of Congress Subject Headings Northwestern University subset (LCH NW), Logical Observation Identifier Names and Codes (LOINC), and National Drug File – Reference Terminology (NDFRT), although the ranking order varies slightly across different datasets. Multimedia Appendix 1 Table-A2 provides example concepts in the top 3 most covered source vocabularies.

**Table 2:** Top 20 mostly covered UMLS source vocabularies.

	Tumblr			Yahoo! Answers								
Rank	Blogs (n=2415)			Questions (n=6452)					Answers (n=10,378)			
	Source vocabulary	# of concepts	%	Source vocabulary		# of concepts		%	Source vocabulary		# of concepts	%
1	SNOMED CT	2315	95.9	SNOMED CT		5476		84.9	SNOMED CT		9032	87.0
2	CHV	1931	80.0	CHV		4928		76.4	CHV		7625	73.5
3	MTH	1774	73.5	MTH		3899		60.4	MTH		5780	55.7
4	NCIt	1156	47.9	MeSH		2957		45.8	MeSH		4796	46.2
5	MeSH	1130	46.8	NCIt		2917		45.2	NCIt		4485	43.2
6	CSP	812	33.6	CSP		1840		28.5	NDFRT		2999	28.9
7	AOD	775	32.1	NDFRT		1775		27.5	CSP		2839	27.4
8	LCH_NW	771	31.9	LCH_NW		1627		25.2	LCH_NW		2436	23.5
9	LOINC	697	28.9	AOD		1585		24.6	AOD		2335	22.5
10	NDFRT	659	27.3	LOINC		1510		23.4	RXNORM		2099	20.2
11	LCH	587	24.3	RXNORM		1421		22.0	LOINC		2081	20.1
12	NCI_NCI-GLOSS	475	19.7	LCH		1187		18.4	LCH		1730	16.7
13	MEDLINEPLUS	402	16.6	NCI_NCI-GLOSS		952		14.8	NCI_FDA		1387	13.4
14	CST	365	15.1	NCI_FDA		868		13.5	DXP		1322	12.7
15	COSTAR	362	15.0	COSTAR		835		12.9	NCI_NCI-GLOSS		1321	12.7
16	NCI_FDA	345	14.3	DXP		830		12.9	COSTAR		1257	12.1
17	OMIM	342	14.2	CST		794		12.3	OMIM		1234	11.9
18	RXNORM	338	14.0	OMIM		790		12.2	CST		1206	11.6
19	DXP	326	13.5	MEDLINEPLUS		721		11.2	VANDF		1117	10.8
20	ICD9CM	241	10.0	VANDF		644		10.0	MTHSPL		1033	10.0

**Table 3:** Top 10 frequently observed concepts covered by both SNOMED CT and CHV.

Rank	Tumblr		Yahoo! Answers			
			Questions		Answers	
	Concept	Freq.	Concept	Freq.	Concept	Freq.
1	Blood (C0005767)	816	Blood (C0005767)	30,654	Blood (C0005767)	54,689
2	Pain (C0030193)	798	Sugars (C0242209)	29,593	Sugars (C0242209)	49,207
3	Insulin (C0021641)	744	Insulin (C0021641)	10,816	Insulin (C0021641)	27,887
4	Pharmaceutical preparations (C0013227)	719	Glucose (C0017725)	7394	Glucose (C0017725)	26,420
5	Sugars (C0242209)	699	Problem (C0033213)	5111	Pharmaceutical preparations (C0013227)	11,571
6	Disease (C0012634)	617	Water (C0043047)	4781	Diseases (C0012634)	9733
7	Problem (C0033213)	568	Pharmaceutical preparations (C0013227)	4456	Carbohydrates (C0007004)	9517
8	Diabetes mellitus (C0011849)	501	Hematologic tests (C0018941)	3784	Problem (C0033213)	9248
9	Teeth structure (C0040426)	424	Pain (C0030193)	3625	Water (C0043047)	5994
10	Operative surgery procedures (C0543467)	375	Urine (C0042036)	2550	Fasting (C0015663)	5848

**Table 4:** Top 10 frequently observed concepts covered by CHV but not SNOMED CT.

	Tumblr		Yahoo! Answers				
Rank			Questions			Answers	
	Concept (CUI)^a^	Freq.	Concept (CUI)		Freq.	Concept (CUI)	Freq.
1	Cider vinegar (C0937941)	54	Stomach (C0038351)		1050	Lantus (C0876064)	689
2	Apple cider vinegar (C1178459)	54	Lantus (C0876064)		571	Actos (C0875954)	659
3	Lantus (C0876064)	15	Humalog (C0528249)		260	Avandia (C0875967)	628
4	Gentle (C0720654)	11	NovoLog (C0939412)		180	HumaLog (C0528249)	289
5	Corrective (C0719519)	9	Glucophage (C0591573)		131	NovoLog (C0939412)	255
6	Botox (C0700702)	9	Levemir (C1314782)		122	Levemir (C1314782)	184
7	RID (C0073361)	6	Actos (C0875954)		95	Glucophage (C1314782)	161
8	HumaLog (C0528249)	5	Seroquel (C0287163)		78	Novolin (C0028467)	112
9	Bead Dosage Form (C0991566)	3	Synthroid (C0728762)		62	Viagra (C0663448)	105
10	Actos (C0875954)	3	Coumadin (C0699129)		54	Triphosphat (C0146894)	77

^a^CUI: concept unique identifier

There was significant overlap between the concepts from the top 2 source vocabularies, SNOMED CT and CHV⎯ 78.2% (1889/2415) concepts from blog postings, 70.0% (4518/6452) concepts in questions, and 68.4% (7095/10,378) concepts in answers. Table 3 shows the top 10 concepts. Note that we only show the preferred term of the concept in the UMLS throughout the paper. Diabetes-related concepts such as *Blood*, *Sugars*, *Insulin*, *Glucose*, and *Diabetes mellitus* were frequently mentioned (preferred names of a UMLS concept are denoted in italics). At the same time, it includes some general medical concepts such as *disease*, *pharmaceutical preparations*, and *problem.* Concepts related to glucose level in blood such as *blood, sugars, glucose,* and *carbohydrates* also appeared with high frequency.

A few concepts were only covered by CHV: 1.7% (40/2415) concepts in blog postings, 6.3% (409/6452) concepts in questions, and 5.1% (529/10,378) concepts in answers. Table 4 shows the top 10 most frequently observed UMLS concepts covered by CHV but not SNOMED CT in our datasets.

All the concepts in Table 4 are about pharmacological substances or organic chemicals, except *stomach* found within questions. Three concepts regarding insulin therapy for diabetes, such as *Lantus* (ie, insulin glargine injection), *Humalog* (ie, insulin lispro injection), and *Actos* (ie, pioglitazone hydrochloride) in blog postings and questions/answers appeared with high frequency. Diabetes-treatment-related concepts, such as *NovoLog* and *Glucophage*, are more frequently observed in questions and answers than blog postings. In total, 9 out of the top 10 concepts in questions and answers were diabetes medications. Only 2 concepts, namely *stomach* in questions and *Viagra* in answers, are not directly related to diabetes treatment. On the other hand, some concepts in blogs were indirectly related to diabetes. For example, *cider vinegar*, *apple cider vinegar*, and *Botox* also frequently appeared.

There were also the concepts covered by SNOMED CT but not CHV: 17.6% (424/2415) concepts from blog postings, 957/6452 (14.8%) concepts in questions and 18.7% (1936/10,378) concepts in answers (See Table 5). Human body related concepts, such as *back structure excluding neck, entire heart*, *entire pancreas*, *entire kidney*, entire *skin,* and *entire eye*, were frequently used to describe their diabetes issues in blog postings or questions/answers. Three concepts, *entire skin*, *symptoms* and *fatty acid glycerol esters* were observed from all our datasets. *Massage* and *training* were frequently mentioned in blog postings, while *injection procedure* and *protective cup* were frequently mentioned in questions and answers but were not mentioned as frequently as in blog postings. As these concepts were frequently observed in social media, CHV should consider importing them to enrich its conceptual content.

**Table 5:** Top 10 frequently observed concepts covered by SNOMED CT but not CHV.

	Tumblr		Yahoo! Answers		
Rank			Questions		Answers	
	Concept (CUI)^a^	Freq.	Concept (CUI)	Freq.	Concept (CUI)	Freq.
1	Entire skin (C1278993)	524	Symptoms (C1457887)	7690	Symptoms (C1457887)	12,727
2	Symptoms (C1457887)	393	Fatty acid glycerol esters (C0015677)	1789	Fatty acid glycerol esters (C0015677)	8727
3	Back structure, excluding neck (C1995000)	236	Entire foot (C1281587)	1647	Entire cells (C1269647)	6435
4	Massage (C0024875)	217	Back structure, excluding neck (C1995000)	1589	Entire heart (C1281570)	3204
5	Fatty acid glycerol esters (C0015677)	210	Entire kidney (C1278978)	1368	Entire pancreas (C1278931)	3003
6	Training (C0220931)	163	Entire eye (C1280202)	1210	Entire skin (C1278993)	2614
7	Entire pancreas (C1278931)	157	Protective cup (C1533124)	1159	Protective cup (C1533124)	2178
8	Entire heart (C1281570)	156	Entire lower limb (C1269079)	985	Entire stomach (C1278920)	1876
9	Entire oral cavity (C1278910)	138	Entire hands (C1281583)	969	Injection procedure (C1533685)	1561
10	Entire spine (C1280065)	137	Entire skin (C1278993)	912	Entire bony skeleton (C1266909)	1501

^a^CUI: concept unique identifier

#### Semantic Types of the Identified Concepts

Among 127 UMLS semantic types (STY), about half of them were identified in our datasets: 52 STYs (40.9%) in the blog postings, 59 STYs (46.5%) in the questions, and 54 STYs (42.5%) in the answers. In general, there was a significant overlap of STYs across our datasets with 52 shared STYs. Seven STYs, however, were identified in the questions only, including “Functional Concept,” “Intellectual Product,” “Laboratory Procedure,” “Organ or Tissue Function,” “Organism Attribute,” “Social Behavior,” and “Substance.” Two STYs, “Fully Formed Anatomical Structure” and “Cell or Molecular Dysfunction,” were not found in questions, but in both the answer dataset and the blog dataset. Table 6 shows the top 20 frequent semantic types of the identified UMLS concepts in the different datasets respectively.

When comparing the top 10 frequently observed STYs across the datasets, 9 out of 10 STYs (ie, “Finding,” “Pharmacologic Substance,” “Therapeutic or Preventive Procedure,” “Disease or Syndrome,” “Organic Chemical,” “Body Part, Organ, or Organ Component,” “Sign or Symptom,” “Medical Device,” and “Amino Acid, Peptide, or Protein”) commonly appeared across the datasets with minor differences in terms of frequency. “Laboratory Procedure” appeared frequently in questions but not in blogs and answers. “Pathologic Function” appeared frequently in answers but not in blogs and questions. Example concepts of the frequently observed STYs showed that laypeople tend to frequently use common concepts to describe their diabetes-related issues in social media. To illustrate, *Sugars*, *Insulin*, *Glucose* ranked in top 5 concepts of the STY “Pharmacologic Substance.” Similarly, the concepts such as *Disease* and *Communicable Diseases* appeared frequently among the concepts of the STY “Disease or Syndrome.” We provide the top 5 frequent concepts for the top 10 frequently observed semantic types in Multimedia Appendix 1 Table A3.

**Table 6:** Top 20 frequently observed semantic types of the identified concepts.

Rank	Tumblr			Yahoo! Answers						
	Blogs			Questions			Answers			
	Semantic type	Concept^a^		Semantic type	Concept		Semantic type	Concept	
		n (%)	Freq.		n (%)	Freq.		n (%)	Freq.
1	Finding	380 (15.7)	5277	Pharmacologic substance	1240 (19.2)	53,976	Pharmacologic substance	1995 (19.2)	185,880
2	Pharmacologic substance	307 (12.7)	4413	Organic chemical	1006 (15.6)	41,255	Organic chemical	1692 (16.3)	123,509
3	Therapeutic or preventive procedure	241 (10.0)	3184	Finding	895 (13.9)	30,458	Disease or syndrome	1511 (14.6)	57,379
4	Disease or syndrome	239 (9.9)	2923	Disease or syndrome	743 (11.5)	28,041	Finding	1302 (12.5)	76,765
5	Organic chemical	225 (9.3)	2737	Body part, organ, or organ component	484 (7.5)	27,172	Body part, organ, or organ component	666 (6.4)	48,584
6	Body part, organ, or organ component	208 (8.6)	2566	Sign or symptom	338 (5.2)	19,601	Therapeutic or preventive procedure	583 (5.6)	16,555
7	Sign or symptom	145 (6.0)	2214	Therapeutic or preventive procedure	331 (5.1)	16,372	Amino acid, peptide, or protein	495 (4.8)	40,521
8	Medical device	134 (5.5)	1319	Amino acid, peptide, or protein	305 (4.7)	13,178	Sign or symptom	436 (4.2)	38,905
9	Amino acid, peptide, or protein	70 (2.9)	1112	Medical device	201 (3.1)	12,862	Medical device	347 (3.3)	20,391
10	Biologically active substance	69 (2.9)	1093	Laboratory procedure	180 (2.8)	10,580	Pathologic function	292 (2.8)	12,551

^a^The percentage was calculated based on the total number of unique identified UMLS concepts: blogs in Tumblr: n=2415, questions in Yahoo! Answers: n=6452, answers in Yahoo! Answers: n=10,378

## Discussion

### Principal Findings

Previous studies [12-15] utilized user-generated documents including social media. However, they mainly used a single test bed based on the assumption that the selected test bed would properly reflect people’s medical concepts. Our study involved different types of social media which contains texts that laypeople generated for different purposes: questions are for expressing their health information seeking needs; blogs and answers are more likely for sharing their knowledge, experiences, and opinions to others. The current study investigated the terminology coverage in consumer-generated text in social media by identifying UMLS concepts and their semantic types. Our findings demonstrated that consumers use medical concepts not only from controlled vocabularies developed for consumers (ie, CHV) but also those for health professionals (eg, SNOMED CT). Our results are in line with prior observations that consumers use both lay and professional terms [24,26,65] and demonstrated that CHV can be enriched by other source vocabularies in the UMLS.

The UMLS concept usage in blogs and social Q&A was different in that the UMLS concepts appeared more frequently in the postings of social Q&A (almost 90% questions and answers) in comparison to blog postings (about 30%). Social Q&A users mainly discuss health-related issues (in the current study, diabetes-related issues) in their postings, because their participation in question asking and answering is purpose-driven. On the other hand, blog users often elaborate nonhealth related topics in their postings, although they tagged their postings with “diabetes.”

In spite of the differences of the overall UMLS concept coverage between blogs and social Q&A, we found that the UMLS concepts identified in different datasets can be covered by a similar number of UMLS source vocabularies. Two UMLS source vocabularies, ie, SNOMED CT and CHV, showed the best coverage. Social media users in our datasets may have advanced medical knowledge because they often use professional terms. CHV demonstrated the second largest coverage for all the datasets despite the fact that CHV has a much smaller number of concepts and terms than SNOMED CT (1:6 ratio). CHV was developed to incorporate consumer expressions presented in consumer-generated text data. Our findings showed that different social media platforms may play a similar role as consumer-generated documents for CHV enrichment, which confirmed the literature [66].

A comparison of the concept coverage between SNOMED CT and CHV in our datasets led us to examine the difference between the concept usages among blog and social media users. For example, *cider vinegar*, *apple cider vinegar*, *massages*, and *training* were frequently mentioned in blog postings, while they were not frequently mentioned in questions and answers. However, concepts pertaining to insulin therapy, such as *Lantus, Humalog*, and *Actos*, are frequently used in questions/answers. Consumers often inquire about a variety of insulin therapies in social Q&A, while blogs often include recipes specific to the use of *vinegar*, a popular ingredient in diabetes-controlling food. *Botox* and *Viagra* were often mentioned in blog postings and answers. They could be important for diabetic patients, although they may not be closely related to control diabetes. It would be interesting to further analyze the relationship of these terms to diabetes. An in-depth analysis of the identified concepts along with how they are used in the original postings could produce useful information for understanding consumers’ information needs and use.

According to our analysis, the percentage of unique concepts covered by CHV but not by SNOMED CT varied from 1.7% to 6.3%. In the blog dataset, where approximately 3000 blogs were analyzed, only 40 concepts were covered by CHV exclusively. On the other hand, in Yahoo! Answers, 409 concepts (6.3%) in questions and 529 concepts (5.1%) in answers were covered by CHV but not by SNOMED CT. These results indicate that the larger datasets would yield more lay concepts. The size of dataset also appeared to affect the diversity of semantics. The same set of 9 semantic types was observed frequently in all our datasets. “Finding,” “Pharmacologic Substance,” and “Disease and Syndrome” were among the top 4 most observed semantic types.

Differences were observed as well. Blogs might be better platforms for consumers to discuss organic chemical, pharmacologic substances, or therapeutic or preventive procedure for diabetes. Yet, concepts of organic chemical and pharmacologic substances were also frequently used in social Q&A. In social Q&A data, 7 semantic types that were not identified in blogs were observed, indicating that larger datasets may yield more diverse medical concepts.

### Limitations

This study has a few limitations. First, the blog data in Tumblr and Yahoo! Answers data were collected in different time frames and are different in size, which might have affected the findings of this study. Smaller volumes of blog data used in this study may affect the diversity of the UMLS concepts identified. Even though blogging and question posting/answering are dynamic online activities for those living with chronic diseases, Tumblr and Yahoo! Answers may not represent all the health information users’ concept usage. The datasets could be expanded to include other types of social media such as diabetes-related discussion boards. The users of these online sources may be biased towards those with greater technological proficiency, such as those who are younger, more educated or those in a higher socioeconomic status who are more likely to seek health information on the Internet. This study may not reflect the experiences of those who are older adults, less educated or underprivileged [67]. Second, even though the automated NLP techniques that were employed in the current study were cost-effective, direct interaction with ordinary health information users would allow the researchers to capture more accurate meaning of medical concepts that these individuals commonly use to describe their health issues. Moreover, a qualitative approach such as content analysis also would help to identify contextual semantics of the concepts. Third, although the WSD function of YTEX is effective in selecting the most possible UMLS concepts for a term in free text, the same term may be matched to different ambiguous UMLS concepts. This is mainly due to the fact that the UMLS may contain unmapped synonymous concepts. Ideally, manual review by domain experts could be applied to further refine the automatic mapping results.

### Conclusions

The current study examined the potential of social media as user-generated documents in which consumers’ medical concepts can be observed and leveraged for controlled vocabulary development for ordinary health information users. We selected and tested 2 social media venues, namely blogs and social Q&A. Our findings showed stronger similarities rather than differences in the controlled vocabulary usage. The size of a dataset may affect the number of concepts identified. However, the similarities in the source vocabularies, frequently used concepts, and semantic types of the concepts indicate that social media sites tend to reflect the common sense of laypeople. More importantly, we found that social media users not only employ consumer concepts in CHV but also concepts in professional vocabularies such as SNOMED CT. This indicates that CHV still has room for improvements by incorporating concepts from other UMLS source vocabularies. The focus of our study is not to identify a list of consumer medical concepts, but to test the feasibility of leveraging social media data to identify consumer concepts covered by existing UMLS source vocabularies. Ultimately, it would assist consumers’ health information searches online, narrowing the disparity between ordinary health information users and medical professionals. In future studies, we will employ automated approaches to identify and recommend new medical terms and concepts from social media to enrich CHV.

## Appendix

Multimedia Appendix 1 Table A1. Full names of the UMLS source vocabularies in Table 2. Table A2. Top 10 frequently observed concepts in the top 3 most covered source vocabularies. Table A3. Top 5 most frequently concepts in the top 9 frequent semantic types.

## Abbreviations

APIs: Application Program Languages
AUI: atom unique identifier
CSP: Computer Retrieval of Information on Scientific Projects Thesaurus
CUI: concept unique identifier
IC: information content
LCH NW: Library of Congress Subject Headings, Northwestern University subset
LOINC: Logical Observation Identifier Names and Codes
MeSH: Medical Subject Headings
NCIt: National Cancer Institute Thesaurus
NDFRT: National Drug File – Reference Terminology
NER: named entity recognition
NLM: National Library of Medicine
NLP: natural language processing
OAC CHV: Open-Access Collaborative Consumer Health Vocabulary
POS: Part-Of-Speech
Q&A: questions and answers
SNOMED CT: SNOMED Clinical Terms
STY: semantic type
UIMA: Unstructured Information Management Architecture
UMLS: Unified Medical Language System
WSD: word sense disambiguation
