# Supplementary material for: Consumers’ Use of UMLS Concepts on Social Media: Diabetes-Related Textual Data Analysis in Blog and Social Q&A Sites
Source: JMIR Med Inform. 2016 Nov 24;4(4):e41. doi: 10.2196/medinform.5748 (PMC5146325; doi:10.2196/medinform.5748)
Supplement: Multimedia Appendix 1 [file medinform_v4i4e41_app1.pdf]

Multimedia Appendix 1

Table A1. Full names of the UMLS source vocabularies in Table 2

Abbreviation of the source vocabulary	Full name	Version
SNOMED CT	US Edition of SNOMED Clinical Terms	March 1, 2015
CHV	Consumer Health Vocabulary	February, 2011
MTH	UMLS Metathesaurus	2015AA
NCIt	National Cancer Institute Thesaurus	2014_03E
MeSH	Medical Subject Headings	January 16, 2015
CSP	Computer Retrieval of Information on Scientific Projects Thesaurus	2006
AOD	Alcohol and Other Drug Thesaurus	2000
LCH_NW	Library of Congress Subject Headings, Northwestern University subset	2013
LOINC	Logic Observation Identifiers Names and Codes	250
NDFRT	National Drug File – Reference Terminology	March 2, 2015
LCH	Library of Congress Subject Headings	1990
NCI_NCI-GLOSS	NCI Dictionary of Cancer Terms	1403E
MEDLINEPLUS	MedlinePlus Health Topics	November 19, 2014
CST	COSTART	1995
COSTAR	COSTAR	1989-1995
NCI_FDA	U.S. Food and Drug Administration	1403E
OMIM	Online Mendelian Inheritance in Man	April 28, 2014
RXNORM	RxNorm Vocabulary	14AB_150302F
DXP	DXplain	1994
ICD9CM	International Classification of Diseases, Ninth Revision, Clinical Modification	2013
VANDF	Veterans Health Administration National Drug File	January 22, 2015

Table A2. Top 10 frequently observed concepts in the top 3 most covered source vocabularies

Tumblr			Yahoo! Answers			
			Questions		Answers	
**Source vocabulary**	**Concept (CUI)**	**Freq.**	**Concept (CUI)**	**Freq.**	**Concept (CUI)**	**Freq.**
SNOMED CT	At risk for impaired home maintenance management (C0231377)	1,438	Blood (C0005767)	26,742	Blood (C0005767)	47,326
	Disease (C0012634)	1,375	Sugars (C0242209)	25,822	Sugars (C0242209)	42,461
	Pharmaceutical Preparations (C0013227)	1,108	Insulin(C0021641)	9,486	Insulin (C0021641)	23,230
	Blood (C0005767)	1,089	Symptoms (C1457887)	7,257	Glucose (C0017725)	23,196
	Heart (C0018787)	1,075	Glucose (C0017725)	6,678	Symptoms (C1457887)	11,908
	Entire heart (C1281570)	1,075	Problem (C0033213)	4,850	Pharmaceutical Preparations (C0013227)	10,657
	Pain (C0030193)	935	At risk for impaired home maintenance management (C0231377)	4,398	Disease (C0012634)	8,774
	Problem (C0033213)	866	Water (C0043047)	4,333	Problem (C0033213)	8,767
	Sugars (C0242209)	712	Pharmaceutical Preparations (C0013227)	4,082	Carbohydrates (C0007004)	8,729
	Asthma (C0004096)	705	Hematologic Tests (C0018941)	3,503	Fatty acid glycerol esters (C0015677)	7,608
CHV	At risk for impaired home maintenance management (C0231377)	1,438	Blood (C0005767)	26,742	Blood (C0005767)	47,326
	Disease (C0012634)	1,375	Sugars (C0242209)	25,822	Sugars (C0242209)	42,461
	Pharmaceutical Preparations (C0013227)	1,108	Insulin (C0021641)	9,486	Insulin (C0021641)	23,230
	Blood (C0005767)	1,089	Glucose (C0017725)	6,678	Glucose (C0017725)	23,196
	Heart (C0018787)	1,075	Problem (C0033213)	4,850	Pharmaceutical Preparations (C0013227)	10657
	Pain (C0030193)	935	At risk for impaired home maintenance management (C0231377)	4,398	Disease (C0012634)	8,774
	Problem (C0033213)	866	Water (C0043047)	4,333	Problem (C0033213)	8,767
	Asthma (C0004096)	712	Pharmaceutical Preparations (C0013227)	4,082	Carbohydrates (C0007004)	8,729
	Operative Surgical Procedures (C0543467)	705	Hematologic Tests (C0018941)	3,503	Water (C0043047)	5,473
	Insulin(C0021641)	678	Pain (C0310367)	3,188	Fasting (C0015663)	5,138
MTH	At risk for impaired home maintenance management (C0231377)	1,438	Blood (C0005767)	26,742	Blood (C0005767)	47,326
	Disease (C0012634)	1,375	Sugars (C0242209)	25822	Sugars (C0242209)	42,461

	Pharmaceutical Preparations (C0013227)	1,108	Level (C2946261)	9893	Insulin (C0021641)	23,230
	Blood (C0005767)	1,089	Insulin (C0021641)	9,486	Glucose (C0017725)	23,196
	Heart (C0018787)	1075	Symptoms (C1457887)	7,257	Level (C2946261)	20,166
	Pain (C0030193)	1,075	Glucose (C0017725)	6,678	Symptoms (C1457887)	11,908
	Problem (C0033213)	935	Problem (C0033213)	4,850	Pharmaceutical Preparations (C0013227)	10,657
	Asthma (C0004096)	866	At risk for impaired home maintenance management (C0231377)	4,398	Disease (C0012634)	8,774
	Operative Surgical Procedures (C0543467)	712	Water (C0043047)	4,333	Problem (C0033213)	8,767
	Level (C2946261)	705	Pharmaceutical Preparations (C0013227)	4,082	Carbohydrates (C0007004)	8,729

[a] Example concepts are the preferred terms in the UMLS.

Table A3. Top 5 most frequently concepts in the top 9 frequent semantic types[a, b]

Tumblr			Yahoo! Answers			
			Questions		Answers	
**Semantic Type**	**Concept (CUI)**	**Freq.**	**Concept* (CUI)**	**Freq.**	**Concept* (CUI)**	**Freq.**
Amino Acid, Peptide, or Protein	Insulin (C0021641)	630	Insulin (C0021641)	9,486	Insulin (C0021641)	23,230
	Proteins (C0033684)	85	Proteins (C0033684)	1,035	Proteins (C0033684)	4,172
	Gluten (C2362561)	18	Lantus (C0876064)	528	Glycosylated hemoglobin A (C0019018)	1,282
	Collagen (C0009325)	13	Properdin (C0033452)	367	Hemoglobin (C0019046)	741
	Glycosylated hemoglobin A (C0019018)	11	Thyrotropin (C0040160)	358	Glucagon (C0017687)	688
Body Part, Organ, or Organ Component	Tooth structure (C0040426)	395	Entire foot (C1281587)	1,482	Kidney (C0022646)	4,208
	Hair (C0018494)	165	Foot (C0016504)	1,393	Liver (C0023884)	2,947
	Entire pancreas (C1278931)	154	Entire kidney (C1278978)	1,195	Entire heart (C1281570)	2,896
	Entire heart (C1281570)	142	Hand (C0018563)	1,192	Entire pancreas (C1278931)	2,797
	Hand (C0018563)	137	Thyroid Gland (C0040132)	1,140	Eye (C0015392)	2,065
Disease or Syndrome	Disease (C0012634)	577	Disease (C0012634)	2,120	Disease (C0012634)	8,774
	Diabetes Mellitus (C0011849)	403	Hypoglycemia (C0020615)	1,384	Communicable Diseases (C0009450)	3,842
	Communicable Diseases (C0009450)	148	Diabetic Retinopathy (C0011884)	1,119	Hypoglycemia (C0020615)	3,734
	Gestational Diabetes (C0085207)	114	Gestational Diabetes (C0085207)	937	Obesity (C0028754)	1,555
	Diabetes Mellitus, Non-Insulin-Dependent (C0011860)	76	Chronic Obstructive Airway Disease (C0024117)	709	Neuropathy (C0442874)	1,410
Finding	At risk for impaired home maintenance management (C0231377)	322	At risk for impaired home maintenance management (C0231377)	4,398	Fasting (C0015663,)	5,138
	Illness (C0221423)	211	Fasting (C0015663)	2,404	Pressure (C0460139)	3,938
	Stress (C0038435)	198	Physical findings (C0311392)	1,845	Systemic arterial pressure (C1272641)	3,428
	Feeling relief (C0564405)	173	Pressure (C0460139)	1,757	Vitality (C0424589)	3,265
	Pressure (C0460139)	160	Systemic arterial pressure (C1272641)	1,511	Body Weight decreased (C0043096)	2,859
Medical Device	Guide device (C0302614)	111	Pump (device) (C0182537)	1,579	Pump (device) (C0182537)	2,152
	Balance, device (C0179199)	91	Protective cup (C1533124)	995	Protective cup (C1533124)	1,752

	Pump (device) (C0182537)	78	Strip medical device (C1321564)	860	Strip medical device (C1321564)	1,571
	Protective cup (C1533124)	54	Insulin pump (C1140609)	834	Spike (C3882441)	1,238
	Pack (physical object) (C1968515)	46	Eyeglasses (C0015421)	632	Test Strip (device) (C2700619)	927
Organic Chemical	Sugars (C0242209)	644	Sugars (C0242209)	25,822	Sugars (C0242209)	42,461
	Glucose (C0017725)	325	Glucose (C0017725)	6,678	Glucose (C0017725)	23,196
	Fatty acid glycerol esters (C0015677)	181	Metformin (C0025598)	1,854	Carbohydrates (C0007004)	8,729
	Oils (C0028908)	129	Fatty acid glycerol esters (C0015677)	1,620	Fatty acid glycerol esters (C0015677)	7,608
	Vitamins (C0042890)	111	Deoxycorticosterone (C0011710)	981	Metformin (C0025598)	2,903
Pharmacologic Substance	Sugars (C0242209)	644	Sugars (C0242209)	25,822	Sugars (C0242209)	42,461
	Pharmaceutical Preparations (C0013227)	642	Level (C2946261)	9893	Insulin (C0021641)	23,230
	Insulin (C0021641)	630	Insulin (C0021641)	9,486	Glucose (C0017725)	23,196
	Level (C2946261)	466	Glucose (C0017725)	6,678	Level (C2946261,)	466
	Glucose (C0017725)	325	Water (C0043047)	4,333	Pharmaceutical Preparations (C0013227)	10,657
Sign or Symptom	Pain (C0030193,)	742	Symptoms (C1457887)	7,257	Symptoms (C1457887)	11,908
	Symptoms (C1457887)	389	Pain (C0030193,)	3,188	Pain (C0030193)	3,341
	Sensory Discomfort (C0234215)	98	Hunger (C0020175)	2,280	Hunger (C0020175)	2,006
	Back Pain (C0004604)	69	Headache (C0018681)	1,894	Fatigue (C0015672)	1,922
	Headache (C0018681)	59	Dizziness (C0012833)	1,256	Headache (C0018681)	1,161
Therapeutic or Preventive Procedure	Therapeutic procedure (C0087111)	341	Injection procedure (C1533685)	728	Injection procedure (C1533685)	1,510
	Interventional procedure (C0184661)	258	Toilet procedure (C0184958)	501	Release (procedure) (C1963578)	754
	Massage (C0024875)	209	Therapeutic tactile stimulation (C0152054)	289	Therapeutic procedure (C0087111)	723
	Chiropractic procedure (C0344217)	190	Diet good (C0452415)	190	Diet good (C0452415)	706
	Physical therapy exercises (C0452240)	101	Contraceptive methods (C0700589)	176	Subcutaneous injection of insulin (C0586328)	601

[a] Example concepts are the preferred terms in the UMLS.
[b] Semantic types are alphabetically ordered.
